# The role of ventilation mode using a laryngeal mask airway during gynecological laparoscopy on lung mechanics, hemodynamic response and blood gas analysis

**Published:** 2016-12

**Authors:** Mohammad Hossein Jarahzadeh, Iman Halvaei, Farshid Rahimi-Bashar, Shekoufeh Behdad, Rouhollah Abbasizadeh Nasrabady, Elahe Yasaei

**Affiliations:** 1 *Anesthesiology and Critical Care Department, Shahid Sadoughi University of Medical Sciences, Yazd, Iran.*; 2 *Department of Anatomical Sciences, Faculty of Medical Sciences, Tarbiat Modares University, Tehran, Iran.*; 3 *Anesthesiology and Critical Care Department, School of Medicine, Hamadan University of Medical Sciences, Hamadan, Iran.*

**Keywords:** *Gynecologic laparoscopy*, *Laryngeal mask airway*, *Volume controlled ventilation*, *Pressure controlled ventilation*

## Abstract

**Background:**

There are two methods for ventilation in gynecological laparoscopy: volume-controlled ventilation (VCV) and pressure-controlled ventilation (PCV).

**Objective:**

To compare the lung mechanics, hemodynamic response and arterial blood gas analysis and gas exchange of two modes of VCV and PCV using laryngeal mask airway (LMA) at different time intervals.

**Materials and Methods:**

Sixty infertile women referred for diagnostic laparoscopy, based on ventilation mode, were randomly divided into two groups of VCV (tidal volume: 10 ml/kg) and PCV. In the PCV group, ventilation was initiated with a peak airway pressure (tidal volume: 10 ml/kg, upper limit: 35 cm H_2_O). In both groups, the arterial blood samples were taken in several time intervals (5, 10 and 15 min after LMA insertion) for blood gas evaluation. Also the lung mechanics parameters were continuously monitored and were recorded at different time intervals.

**Results:**

There were no significant differences for patient’s age, weight, height and BMI in two groups. The peak and plateau airway pressure were significantly higher in VCV group compared to PCV group 5 and 10 min after insertion of LMA. PaO_2_ was significantly higher after 10 and 15 min in VCV group compared to PCV group (p=0.005 and p=0.03, respectively). PaCO_2_ showed significant increase after 5 min in PCV group, but the differences were not significant after 10 and 15 min in two groups. The end tidal CO_2_ showed significant increase after 10 and 15 min in VCV compared to PCV group.

**Conclusion:**

Both VCV and PCV seem to be suitable for gynecological laparoscopy. However, airway pressures are significantly lower in PCV compared to VCV.

## Introduction

Gynecological laparoscopy for diagnosis and surgery is performing increasingly worldwide. It may be associated with cardiorespiratory effects via pneumoperitoneum and systemic resorption of carbon dioxide (CO_2_) via peritoneal surfaces (1). There are two methods for ventilation: volume-controlled ventilation (VCV) and pressure-controlled ventilation (PCV). In VCV, as a conventional mode, the flow is constant. PCV, as an alternative mode, is proposed for improving oxygenation in the intensive care unit, adult respiratory distress syndrome cases, obese patients undergoing bariatric surgery, and one-lung ventilation (2-4). Because of specific position during gynecological laparoscopy, the diaphragm moves up and may lead to decrease compliance and barotrauma (5). Improving ventilation and selecting the best method of ventilation would be worthwhile for these groups of patients. During gynecological laparoscopy with Trendelenberg position that the ventilation will be decreased due to pressure limitation, PCV improves ventilation by increasing the rate of tidal volume compared to VCV (6).

The laryngeal mask airway (LMA) was introduced decades ago as an alternative method when endotracheal intubation is not necessary (7). LMA is suggested to be used in patients undergoing laparoscopic surgery (5, 8-10). There is not general agreement about the best mode of ventilation for gynecological laparoscopy surgeries. It has been suggested that PCV is superior to VCV using LMA in children (11). Jeon *et al* proposed that PCV may be more efficient compared to VCV in gynecological laparoscopy (5). 

To the best of our knowledge, few studies have compared cardiopulmonary and respiratory mechanics, hemodynamic and gas exchange parameters between VCV and PCV in diagnostic gynecological laparoscopy using LMA. Our main goal was to compare the lung mechanics, hemodynamic response and arterial blood gas analysis and gas exchange of two modes of VCV and PCV using LMA at different time intervals.

## Materials and methods

Sixty women who were electively referred for diagnostic laparoscopy due to infertility were entered in this cross-sectional study. Informed written consent was obtained from all patients. This study was approved by ethic committee of Shahid Sadoughi University of Medical Sciences, Yazd, Iran.

The patients with history of reflux, airways anomalies and difficult intubation were excluded from the study. American Society of Anesthesiologists (ASA) physical status of patients was class I and II. The patients were randomly divided into two groups of VCV and PCV based on ventilation mode. Ventilation mode was randomly selected for the patients and operating room, recovery room staffs and laboratory technicians were blinded for the type of ventilation. 

The baseline values for the systolic arterial pressure (SAP), mean arterial blood pressure (MAP), heart rate and oxyhemoglobin saturation measures by puls oxymeter were recorded firstly. After insertion of a 20G i.v. cannula, anesthesia was induced with propofol 2.5 mg/kg. Atracurium 0.5 mg/kg was used as a muscle relaxant. For analgesia, fentanyl 100 µg was administrated and isofluran was used for maintenance of anesthesia. After preoxygenation with 100% O_2_, the LMA-classic was inserted by an expert anesthesiologist. Nosogastrial tube 18 was used for all patients and was removed after suction of gastric fluids. Mechanical ventilation was performed with an Avance (Prima, UK). After completion of the surgery, the residual neuromuscular block was reversed with neostigmine 0.05 mg/kg and atropine 0.025 mg/kg.

In the VCV group, ventilation was performed with a tidal volume of 10 ml/kg body weight. The respiratory rate was considered to be 12 breaths/min to adjust end tidal volume carbon dioxide pressure in a normal range. The inspiratory/expiratory ratio was set at 1:2. In the PCV group, ventilation was initiated with a peak airway pressure with a tidal volume of 10 ml/kg (upper limit: 35 cmH_2_O). In both groups, the blood samples were taken from the radial artery in several time intervals for blood gas evaluation. The first sample (T1) was taken 5 min after insertion of LMA. 

The second (T2) and third samples (T3) were taken after 10 and 15 min, respectively. Also the compliance, airway resistance, end tidal volume, peak airway pressure, plateau airway pressure, SAP, MAP, heart rate, arterial oxygen pressure, arterial CO_2_ pressure, end-tidal CO_2_, and arterial oxygen saturation were continuously monitored during the anesthesia and were recorded at 5, 10 and 15 min after LMA insertion. 

**Statistical analysis**

The data are presented as mean±SD for numerical data and percentage for categorical values. Independent sample t-test was applied for comparison numerical data between two groups. Chi-square and Fisher exact test were used for comparison of qualitative data between two groups. p<0.05 was considered to be statistical significant.

## Results

There were no significant differences for patient’s age, weight, height and body mass index (BMI) in two groups (Table I). The compliance, airway way resistance and end tidal volume had no significant differences in two groups, 5, 10 and 15 min after insertion of LMA (Table II). The peak and plateau airway pressure were significantly higher in VCV group in comparison with PCV group after 5 and 10 min insertion of LMA. Patient’s hemodynamic responses were similar in both groups after different time intervals. PaO_2_ was significantly higher after 10 and 15 minutes in VCV group in comparison with PCV group (p=0.005 and p=0.03, respectively, Table III). Although PaCO_2_ showed significant increase after 5 min in PCV group, but the differences were not significant after 10 and 15 min in two groups. The end tidal CO_2_ showed significant increase after 10 and 15 min in VCV compared to PCV group.

**Table I:** Patient demographics in two groups

**Variables**	**Groups**		**p-value**
	**VCV**	**PCV**	
Age (yr)	27±8.2	31.6±8.3	0.1
Weight (kg)	62.9±8.9	63.9±6.7	0.1
Height (cm)	163.2±7.7	160.4±5.5	0.1
BMI (kg/m^2^)	23.6±3.5	24.8±2.7	0.1

Values are presented as mean ± SD

VCV: volume-controlled ventilation

PCV: pressure-controlled ventilation

BMI: body mass index

**Table II:** Comparison of lung mechanics and hemodynamic response at different time intervals in two groups

**Variables**	**Time intervals**											
	**T1**			**T2**				**T3**				
	**VCV**	**PCV**	**p-value**		**VCV**	**PCV**	**p-value**		**VCV**	**PCV**	**p-value**	
Compliance (ml/cmH2O)	50.7±13.8 55.5 (26.8-66.2)	84.3±130.6 54.2 (25.5-662)	0.1	34.6±6.8		34.5±7	0.2	34±8.2 31 (24.7-46.6)		34.1±7.7 30.6 (23.6-56.9)		0.2
Airway resistance (cmH2O/L/s)	3.5±0.8	3.5±0.7	0.6	3.4±0.6		3.03±0.06	0.3	3.5±0.8		3.5±0.7		0.6
End tidal volume (ml)	533.9±49.6	504.8±108.8	0.09	543.2±77.8		498.8±123.1	0.053	556.1±95.2		585.1±128.9		0.5
Peak airway pressure (cmH2O)	22.2±27.3 15.5 (13-160)	14.6±4 13 (10-27)	<10^-4^	23.4±3 24.5 (18-28)		20±3 19 (14-27)	<10^-4^	23±3.6 23 (18-35)		22.8±4 12 (14-29)		0.4
Plateau airway pressure (cmH2O)	16±3.9 14.5 (13-26)	14±4 12 (11-26)	<10^-4^	22.2±3 23 (17-27)		19±3 18 (13-26)	<10^-4^	21.5±2.6 22 (17-26)		21.9±4 23 (13-28)		0.2
SAP (mmHg)	102.9±11.6	109.1±12.9	0.06	125.7±14.5		126.8±19.5	0.2	113.5±14.8		121.8±19.2		0.3
MAP (mmHg)	75.5±11.1	85.2±18.2	0.1	101.4±12		100.5±14.6	0.9	88.5±10.8		95.3±15.3		0.2
HR (bpm)	82±13.3	84.6±18.8	0.2	85.8±12.4		83.8±13.9	0.2	83.8±16.2		84.9±13.8		0.1

Values are presented as mean ± SD, median (min-max).

T1: 5 min after laryngeal airway insertion

T2: 10 min after laryngeal airway insertion

T3: 15 min after laryngeal airway insertion,

VCV: volume-controlled ventilation

PCV: pressure-controlled ventilation

SAP: systolic arterial pressure

MAP: mean arterial pressure

HR: heart rate

**Table III:** Arterial blood gas analysis and gas exchange at different time intervals in two groups

**Variables**	**Time intervals**									
	**T1**			**T2**			**T3**			
	**VCV**	**PCV**	**p-value**	**VCV**	**PCV**	**p-value**		**VCV**	**PCV**	**p-value**
PaO_2_	383.4 ± 53.3	317.6 ± 119.5	0.1	369.9 ± 88	296.7 ± 110.1	0.005		368.7 ± 58.7	317.9 ± 113.6	0.03
PaCO_2_	31.2 ± 4.1	33.3 ± 7.1	0.04	35.3 ± 4.8	40.1 ± 12.1	0.06		35.8 ± 7.4	37.9 ± 8.9	0.6
EtCO_2_	33.2 ± 2.9	35.1 ± 2.9	0.08	37.3 ± 2.9	36.3 ± 3.1	0.04		39.9 ± 4.9	35.8 ± 3	0.001
SaO_2_	99.9 ± 0.3	99.8 ± 0.8	0.7	99.6 ± 0.9	99.8 ± 0.4	0.5		99.7 ± 0.6	99.9 ± 0.2	0.1
pH	7.44 ± 0.02	7.39 ± 0.05	0.002	7.39 ± 0.04	7.22 ± 0.62	0.01		7.37 ± 0.06	7.08 ± 0.86	0.3

Data are means ± SDs.

T1: 5 min after laryngeal airway insertion

T2: 10 min after laryngeal airway insertion

T3: 15 min after laryngeal airway insertion

VCV: volume-controlled ventilation

PCV: pressure-controlled ventilation

PaO_2_: partial pressure of oxygen in the arterial blood

PaCO_2_: partial pressure of carbon dioxide in the arterial blood

EtCO_2_: end-tidal carbon dioxide, SaO2: O_2_ saturation.

## Discussion

Introducing the most efficient method of ventilation has been a matter of research during last decades. VCV method as a routine mode is applied for ventilation in different surgeries. In laparoscopic surgeries, because of difficult conditions like pneumoperitoneum and Trendelenburg position, respiratory mechanics would be impaired. There are several studies regarding introducing an alternative method for VCV. We, for the first time, compared the parameters of arterial blood gas analysis at three different time interval after LMA insertion. 

In this study we tried to evaluate the efficacy of PCV method, as an alternative method, compared to VCV at different time intervals. Our results showed that peak airway pressure would be lower in PCV mode compared to VCV mode and risk of baro-trauma is reduced in PCV mode. 

It has been shown that LMA can produce enough ventilation as a good choice for laparoscopic surgeries (8-9). It is reported that LMA-classic can reduce and maintain peak airway pressures in pneumoperitoneum in comparison with Cobra perilaryngeal airway (9). In laparoscopic gynecologic surgeries pneumoperitoneum and the Trendelenburg position are two main reasons that are also responsible for increasing peak airway pressure because in this situation, the diaphragm makes pressure on the lungs and therefore residual capacity and compliance decrease (12). 

One of the main advantages of PCV is reduction in peak airway pressure due to low inspiratory flow, so consequently this mode can minimize the lung traumas following increasing peak and plateau air pressure. Our data showed that both peak and plateau airway pressure were significantly lower in PCV group in comparison with VCV 5 and 10 min after ventilation with LMA which was in line with previous findings (1, 13-14). 

Jeon *et al* compared VCV and PCV in laparoscopic surgeries 5 min after LMA and 5 and 15 min after CO_2_ insufflation. They found that peak airway pressure 5 and 15 min after CO_2_ insufflation was significantly higher in VCV group compared to PCV group. Also they found that compliance was decreased and airway resistance was significantly higher 5 and 15 min after CO_2_ insufflation in both groups of VCV and PCV (5). 

Our data showed that there are no differences for compliance and airway resistance between two groups 5, 10 and 15 min after LMA. Also our data revealed that peak airway pressure were significantly lower in PCV group in comparison with VCV group 5 and 10 minutes after LMA. 

Jeon *et al* found that the PaCO_2_ 5 minutes after LMA and 15 min after CO_2_ insufflation were significantly lower in PCV group compared to VCV group. They reported that PaO_2_ was lower 15 min after CO_2_ insufflation compared to 5 min after LMA insertion in both groups (5). But our data showed that PaO_2_ was significantly higher in VCV compared to PCV 10 and 15 min after LMA. However, EtCO_2_ was significantly higher in VCV compared to PCV 10 and 15 min after LMA. Our data also showed that there were no significant differences for compliance between two groups which was in line with previous findings (5).

Our results showed some parameters of arterial gas analysis are in favor of VCV and some are in favor of PCV. VCV keeps adequate tidal volume and CO_2_ can be properly eliminated in this mode while it was shown that PCV makes the gas distribution more homogenous and better ventilation-perfusion matching could be anticipated (15-16). 

There is a similar study comparing PCV and VCV in one lung ventilation for thoracic surgery. It was shown that there is no difference for oxygenation between PVC and VCV in patients with good pulmonary functions (17). In another study, it was shown that the respiratory mechanics and gas exchange are the same between PCV and VCV (18).

## Conclusion

In conclusion, it seems that PCV is a good alternative for patients undergoing gynecological laparoscopy in order to reduce airway pressures. More studies are required for comparing the VCV and PCV on different parameters of lung ventilation especially gas exchange and arterial blood gas for longer times.

## Note

***This article extracted from residential thesis. (Elahe Yasaei)***
